# Development of Low-Pressure Die-Cast Al−Zn−Mg−Cu Alloy Propellers—Part Ⅰ: Hot Tearing Simulations for Alloy Optimization

**DOI:** 10.3390/ma17133133

**Published:** 2024-06-26

**Authors:** Min-Seok Kim, Jiwon Kim

**Affiliations:** 1 Department of Materials Science and Engineering, Gachon University, Seongnam-si 13120, Republic of Korea; 2 Materials Science and Chemical Engineering Center, Institute for Advanced Engineering, Yongin-si 17180, Republic of Korea; jkim@iae.re.kr

**Keywords:** Al−Zn−Mg−Cu alloy, low-pressure die casting, simulation, propeller, hot tearing

## Abstract

Recent advances in the leisure boat industry have spurred demand for improved materials for propeller manufacturing, particularly high-strength aluminum alloys. While traditional Al-Si alloys like A356 are commonly used due to their excellent castability, they have limited mechanical properties. In contrast, 7xxx series alloys (Al−Zn−Mg−Cu based) offer superior mechanical characteristics but present significant casting challenges, including hot-tearing susceptibility (HTS). This study investigates the optimization of 7xxx series aluminum alloys for low-pressure die-casting (LPDC) processes to enhance propeller performance and durability. Using a constrained rod-casting (CRC) method and finite element simulations, we evaluated the HTS of various alloy compositions. The results indicate that increasing Zn and Cu contents generally increase HTS, while a sufficient Mg content of 2 wt.% mitigates this effect. Two optimized quaternary Al−Zn−Mg−Cu alloys with relatively low HTS were selected for LPDC propeller production. Simulation and experimental results demonstrated the effectiveness of the proposed alloy compositions, highlighting the need for further process optimization to prevent hot tearing in high Mg and Cu content alloys.

## 1. Introduction

Recent advances in the leisure boat industry have led to rapid growth in the market for related components and materials [1,2,3,4,5]. Particularly, there has been a surge in demand for small propellers used in boat propulsion [2,3,4,5,6]. Currently, most propellers for leisure boats are manufactured using stainless-steel or aluminum alloys (Al-Si alloys) for casting [7,8]. Stainless-steel propellers are widely utilized in boats requiring high performance due to their excellent corrosion resistance and mechanical properties [8]. However, their high melting point makes casting challenging, resulting in significantly higher product costs compared to aluminum propellers [9]. On the other hand, propellers made from Al-Si alloys offer the advantage of lightweight design and approximately one-third lower product costs compared to stainless-steel products [9,10]. Nonetheless, they exhibit limitations in mechanical properties, impacting boat performance and durability. To address these limitations, there is a demand for the development of new metallic materials, such as high-strength aluminum alloys, for propeller manufacturing [9].

Traditional Al-Si alloys like A356 exhibit excellent castability but have limitations in improving mechanical properties. In contrast, 7xxx series alloys (Al−Zn−Mg−Cu-based alloys) represent high-strength aluminum alloys with outstanding mechanical characteristics [11]. These alloys demonstrate superior mechanical properties not only in the as-cast state but also offer significant potential for strength enhancement through proper alloy design and heat treatment processes [11]. Therefore, applying 7xxx series alloys makes it feasible to develop products surpassing the mechanical properties of conventional stainless-steel products, making them viable alternatives. However, one crucial consideration is the issue of corrosion resistance in 7xxx series alloys [12,13]. Fortunately, for leisure boat propellers, which are consumables, it is presumed that this issue can be somewhat mitigated through surface treatments such as anodizing when moving away from Al-Si alloys [14,15]. With this premise in mind, research and development efforts are aimed at overcoming this challenge.

Conventional 7xxx series aluminum alloys were originally developed for wrought applications, posing challenges for their utilization as casting materials. The wide temperature range in solidification and higher shrinkage during cooling compared to Al-Si alloys necessitate higher casting complexity [16]. Furthermore, the relatively low eutectic liquid fraction at the final solidification region exacerbates sensitivity to shrinkage porosity and hot tearing during casting [17]. Therefore, for the development of casting materials, it is imperative to modify and optimize alloy compositions to enhance castability rather than directly applying commercially available high-strength alloys [18]. Another fortunate aspect in this regard is that the minor alloying elements such as Mg and Cu can vary without significantly compromising the superior mechanical properties compared to conventional Al-Si alloys. Therefore, there is less concern about the burden of extensive compositional changes. Consequently, alloy modifications focusing primarily on castability are feasible as a primary consideration.

Most conventional leisure boat propellers are manufactured using traditional gravity-casting processes [19]. While cost-effective, these gravity-casting processes have limitations in controlling casting defects such as internal shrinkage voids or gas porosity [20]. Particularly for the propeller products, the coexistence of thick hub sections and thin blade areas can lead to casting defects such as incomplete melt filling (i.e., misrun) or hot tearing due to abrupt thickness variations in the casting and sharp angle changes between the hub and blades. Low-pressure die-casting (LPDC) processes typically involve supplying molten metal from below, minimizing turbulence in the molten metal flow, and are widely used for high-quality casting production [21]. By applying this process, a stable molten metal supply and improved cooling rates can effectively control internal casting defects.

**Figure 1:**
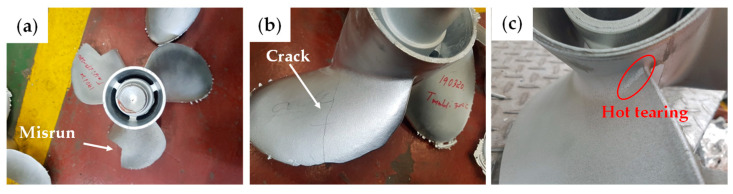
Examples of casting defects in the LPDC 7xxx series aluminum alloy propellers: (**a**) misrun; (**b**) crack; (**c**) hot tearing.

Figure 1 shows an example of casting defects occurring during the production of propeller products using 7xxx series alloys in a mass-production line of the LPDC process. Various casting defects, including misrun and cracks in the blade area, were observed in the initial casting tests of the 7xxx series alloy. Particularly noteworthy were the hot tears occurring at the connection points between the hub and blades, which were not easily remedied through on-site process improvements. This indicates that both alloy and process optimization are necessary from the perspective of controlling hot tearing to produce 7xxx series alloy products.

To optimize the alloy for propeller products by applying a completely new alloy rather than just adjusting minor alloying elements in conventional commercial alloys, extensive alloy composition testing and process optimization are required. In this case, the most effective approach is the application of numerical simulation technology. As shown in previous studies, predicting hot cracking susceptibility (HTS) using simulation models has shown to be quite effective [22,23,24,25,26]. This study aims to evaluate the HTS of various commercially available 7xxx series aluminum alloys and develop a simulation model for predicting HTS based on these results. From a practical perspective, simulations are conducted using widely validated commercial software [22]. Utilizing the simulation model, this study investigates the effect of compositional changes in each component in Al−Zn−Mg−Cu alloys on HTS and conducts optimization to secure castability.

## 2. Materials and Methods

### 2.1. Casting Process for Evaluating Hot Tearing in Commercial Aluminum Alloys

**Figure 2:**
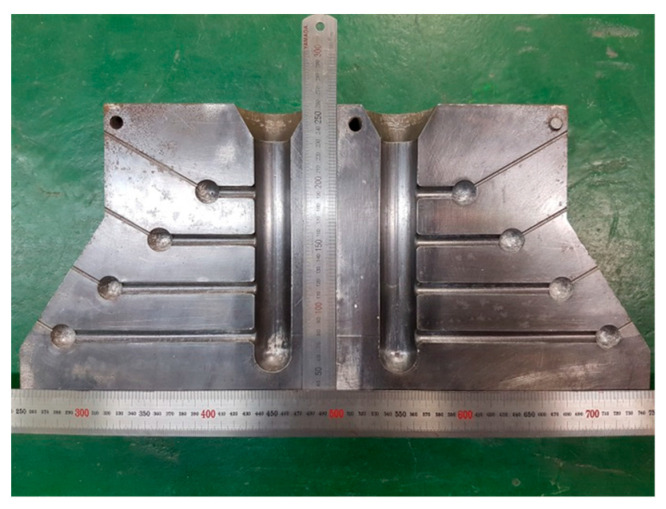
Appearance of mold for constrained rod casting.

To evaluate the HTS of Al−Zn−Mg−Cu alloys and optimize the simulation model, casting was conducted for various commercial alloys. The alloys and their compositions used in the casting process are summarized in Table 1. The chemical composition of each alloy was analyzed using the ICP-OES (inductively coupled plasma optical emission spectrometry) analysis method. Casting was performed using the constrained rod-casting (CRC) process to assess the HTS of each alloy. Figure 2 illustrates the appearance of the mold used for CRC. H13 steel was utilized as the mold material, consisting of two fully symmetrical pieces joined together. The mold features four types of rod cavities with varying lengths. The lengths of the rods are, in ascending order, 51 mm, 89 mm, 127 mm, and 165 mm, respectively, while the diameter of the rod cavities remains consistent at 95 mm. The castings were carried out under identical initial melt temperature conditions with a melt superheat of 100 °C, considering the liquidus temperature of each alloy. The initial mold temperature was set to 150 °C.

Based on the CRC results, the HTS was evaluated. The severity of hot tearing can be categorized into four levels: short hairline, full hairline, crack, and half-broken rod. Based on visual inspection of these four cases, the HTS can be calculated using the following equation [26]:

(1) $$HTS=\sum f_{crack}f_{length}f_{location}$$

where *f_crack_* is the factor for the crack severity, *f_length_* is the factor for the rod length, and *f_location_* is the factor for the crack location. The values of each factor are summarized in Table 2.

### 2.2. Numerical Simulation Models

#### 2.2.1. Simulation Model for Predicting Hot Tearing

A finite element simulation was conducted to predict the HTS of the casting process. For the simulation, the commercial software ProCAST 2021 was utilized to perform coupled thermal–fluid–stress analysis. ProCAST software provides a hot-tearing indicator (HTI) module based on Gurson’s constitutive model, specifically designed for predicting hot tearing in the casting process. This HTI is a strain-driven model that utilizes the total strain accumulated during solidification. It is defined by accumulated plastic strain in the mushy zone that corresponds to the void nucleation described in the Gurson Model [22]:

(2) $$HTI=\int_{t_{c}}^{t}\sqrt{\frac{2}{3}{\dot{\varepsilon }}^{P}:{\dot{\varepsilon }}^{P}}d\tau ,\;t_{C}\;\leq t\;\leq \;t_{S}$$

where ${\dot{\varepsilon }}^{P}$ is the effective plastic strain rate, $t_{C}$ is the time at the coherency temperature, and $t_{S}$ is the time at the solidus temperature. To evaluate the HTS values for each load based on the CRC results used in this study, HTS_simul._ values were derived using the criteria from Table 2. For HTS_simul._ calculations, the HTI values obtained from simulations were employed instead of the *f_crack_* values from Table 2. As for the *f_location_* calculations for HTS_simul._, only cases where *f_location_* = 1 and 2 were used, considering the accumulated strain occurring at the sprue and ball regions in the simulation.

#### 2.2.2. Model Geometry and Mesh

**Figure 3:**
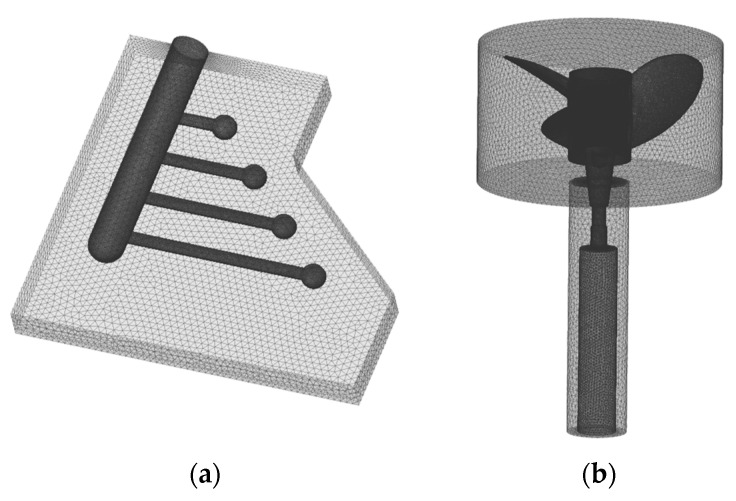
Simulation models showing 3D mesh: (**a**) CRC; (**b**) LPDC.

Figure 3 depicts the simulation model and mesh for the constrained rod mold and the LPDC process for propellers. In the CRC model (Figure 3a), the geometry was modeled to match the actual mold size, and the mesh was configured differentially, considering the importance of the analysis part and calculation time. The mesh size was set to 1 mm for the casting area and 5 mm for the mold. The LPDC model was constructed to reflect the actual in-plant process from the stoke part in the melt container to the cooling mold part, maintaining the same external dimensions as the actual caster. The mesh was differentially configured, and for the propeller section where solidification occurs, a finer mesh of approximately 2 mm average size was employed.

#### 2.2.3. Material and Casting Parameters for Simulation

**Figure 4:**
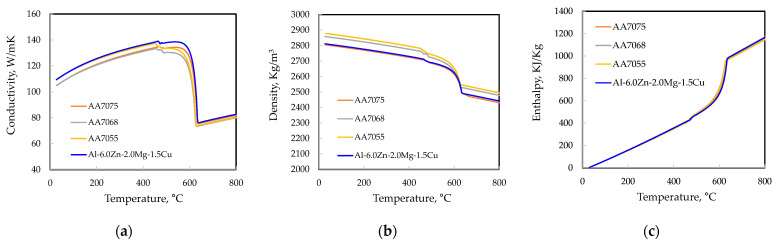

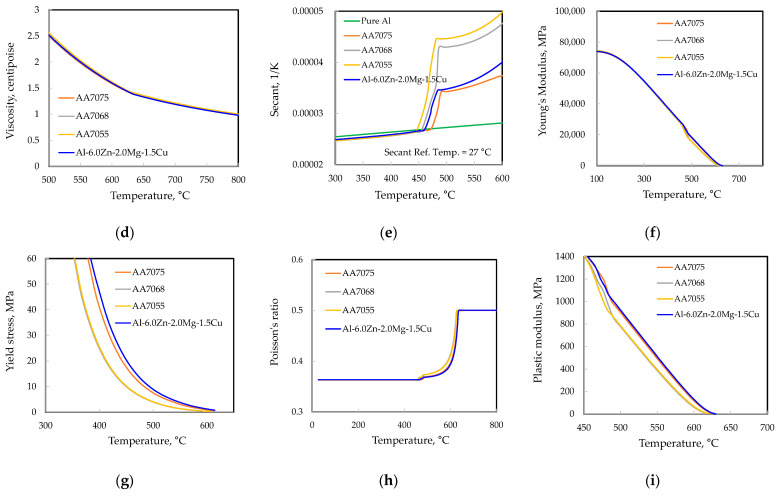
Thermo-physical properties of aluminum alloys used in the simulation: (**a**) conductivity; (**b**) density; (**c**) enthalpy; (**d**) viscosity; (**e**) thermal expansion; (**f**) Young’s modulus; (**g**) yield stress; (**h**) Poisson’s ratio; (**i**) plastic modulus.

Figure 4 illustrates the thermo-physical properties of aluminum alloys and components in the LPDC process employed in the numerical simulation. The material properties were obtained using the database of PanAl2021 software, which is widely used in ProCAST to acquire material properties for simulation models [22]. The composition of the aluminum alloy for simulation was calculated using the nominal composition from Table 1. The properties of the alloy were calculated using the back diffusion cooling condition [27]. When calculating the properties of an alloy considering back diffusion, the cooling rate is an essential factor. It plays a crucial role in determining the solidification temperature range (STR) and the volume fraction of the eutectic phase, *f_eutectic_*, in the alloy. As summarized in Table 3, the experimentally obtained *f_eutectic_* from various references [28,29] were compared with simulation results under different cooling rates to derive appropriate cooling rate conditions. The comparative analysis reveals a clear finding: for all alloys, the Scheil cooling condition significantly overestimated the actual volume fraction of the eutectic phase. This indicates that applying back diffusion is more effective for obtaining a more accurate volume fraction of the eutectic phase. Under back diffusion conditions, the calculation results showed that as the total alloy composition increased, the lower cooling rate conditions more closely resembled the experimental data. In this study, considering the current range of alloy compositions, calculations were conducted at a cooling rate of 20 K/s.

The simulation for the CRC process was conducted under the same conditions as the actual casting conditions mentioned above. The LPDC process simulation was carried out to reflect production conditions in the mass-production line, and the initial and boundary conditions are summarized in Table 4.

## 3. Results and Discussion

### 3.1. Hot-Tearing Susceptibility of Commercial 7xxx Alloys in Constrained Rod Castings

**Figure 5:**
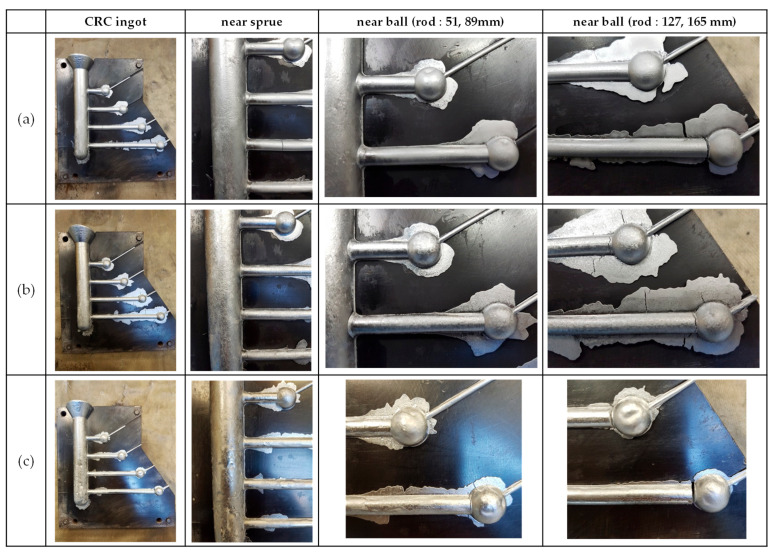
Results of constrained rod casting: (**a**) AA7075; (**b**) AA7068; (**c**) AA7055.

Figure 5 shows the results of constrained rod casting for AA7075, AA7068, and AA7055 alloys. To calculate the experimental hot-tearing susceptibility (HTS_exp._) values, careful evaluation was conducted with the molds left inserted to prevent the widening of cracks upon separation of the casting material from the mold. Cracks were primarily observed at the sprue and ball, with some occurring at the midpoint of certain rods. The severity of cracks increased with the length of rods in each alloy, and the total HTS_exp._ values were observed to be 328, 376, and 384 for the AA7075, AA7068, and AA7055 alloys, respectively.

To identify the factors influencing the trend of HTS_exp._ in CRC alloys, the variation in solid fraction with temperature for each alloy was calculated. Various factors potentially affecting hot tearing were summarized in Table 5 based on the calculation, considering back diffusion cooling conditions. These factors include the total solidification temperature range from liquidus to solidus ($∆T_{total})$, the solidification temperature range within the condition of 0.9 < fs < 0.98 ($∆T_{0.9-0.98})$, and $∆T_{0.9-0.98}$/$∆T_{0.4-0.9}$ according to the Clyne and Davies’ model [30], the volume fraction of the eutectic phase at the final solidification region ($f_{eut.})$ and the solidification temperature range of the eutectic phase ($∆T_{eut.})$. The results of comparing the experimental HTS_exp._ values with the factors in Table 5 revealed a tendency for the HTS_exp._ values to increase as both $∆T_{total}$ and $∆T_{eut.}$ increased. However, the other factors did not exhibit a discernible trend.

Upon evaluating the HTS of commercial 7xxx series alloys through CRC, it is evident that the trends align well with previous research findings: HTS tends to increase with increasing Zn and Cu contents, while it decreases to some extent with increasing Mg content [26,28]. This is closely associated with the ability of the residual liquid to effectively fill the gaps between the grains formed by solidification shrinkage and thermal contraction during the final solidification stage of the alloy. Clyne and Davies [30] suggested that the susceptibility to hot cracking increases as the vulnerable period where 0.9 < f_s_ < 0.98 spent in the hot cracking region (t_v_), the total solidification temperature range of the alloy (∆T), and the grain size increase. Conversely, they proposed that the susceptibility decreases as the time available for stress relief (t_R_), where sufficient liquid feeding can take place during solidification, increases. In multi-component alloys, the hot-tearing mechanism becomes more complex. J.H. Kim et al. [29] demonstrated that the solidification temperature range, grain size, and eutectic phase fraction collectively determine the HTS in Al−Zn−Mg−Cu alloys with various Zn, Mg, and Cu compositions. They observed that, in cases with a relatively low fraction of eutectic phases, HTS tends to decrease as the STR decreases and the eutectic phase fraction increases. However, when a sufficient amount of eutectic phase is formed, sufficient liquid feeding at the final solidification stage can significantly improve HTS, regardless of the STR. The influence of the STR is more pronounced for Mg and Cu alloys than for Zn alloys, while the fraction of the eutectic phase shows an increasing trend with increasing Zn, Mg, and Cu content. In this study, under given casting conditions of CRC (i.e., a constant cooling rate) and non-grain refined conditions, the STR of the alloy is ultimately considered a critical factor in determining HTS. The alloys with higher alloying contents, such as AA7068 and AA7055, exhibited relatively high eutectic phase fractions. This can facilitate liquid feeding to some extent, which may help reduce the HTS. However, it is inferred that the increase in STR, particularly due to the increase in the temperature range in which the eutectic phase solidifies ($∆T_{eut.})$, led to an increase in HTS_exp._ values.

### 3.2. CRC Process Simulation for HTS Evaluation

**Figure 6:**
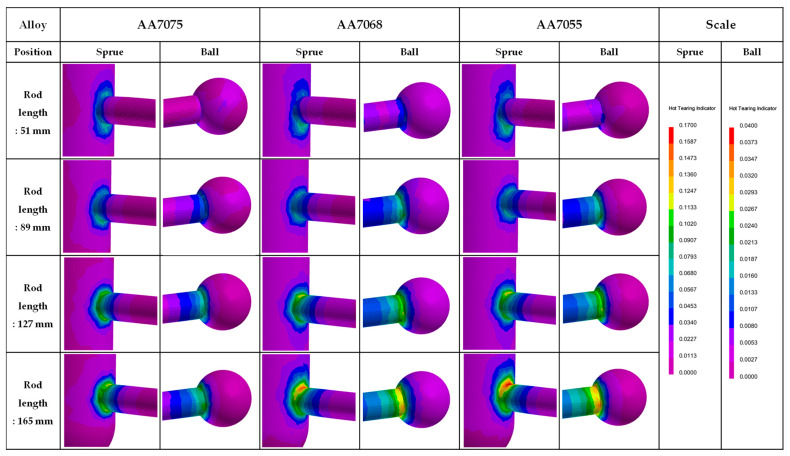
Simulation results of the hot-tearing indicator for CRC 7xxx alloys.

Figure 6 presents the simulation results predicting the hot-tearing indicator (HTI) in the CRC process of commercial alloys. Relatively high HTI values were observed near the sprue and ball for each alloy, and there was a tendency for the maximum HTI value in each rod to increase as the length of the load increased. When comparing alloys, the HTI values increased in the order of AA7075 < AA7068 < AA7055 for the same rod position. By utilizing the maximum HTI values in each part and Table 2 index, the HTS_simul._ values were calculated, resulting in values of 9.7, 11.8, and 12.4 for the AA7075, AA7068, and AA7055 alloys, respectively.

**Figure 7:**
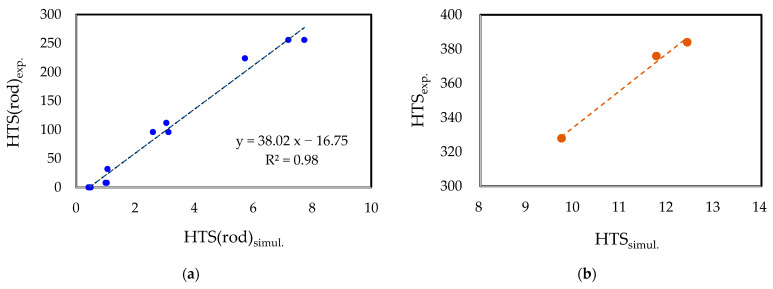
Comparison of simulation and experiment results for CRC 7xxx alloys: (**a**) HTS(rod)_exp._ vs. HTS(rod)_simul._; (**b**) HTS_exp._ vs. HTS_simul._.

To further compare the simulation results with the CRC experiment results, the HTS values for each rod (HTS(rod)) were analyzed. Figure 7a exhibits the relationship between the HTS(rod)_simul._ and HTS(rod)_exp._ values for each alloy and rod. The comparison revealed a well-established linear relationship between the predicted HTS(rod)_simul._ values and the experimentally evaluated HTS(rod)_exp._ values. This suggests that the HTS(rod) values for each rod are reasonably predictive of the overall HTS, indicating a high level of reliability in predicting the HTS values. Figure 7b illustrates the comparison between the total HTS values obtained from the simulation and the experimental results. It demonstrates a noticeable trend wherein the HTS_exp._ values increase with an increase in the HTS_simul._ values, thus effectively capturing the overall trend.

### 3.3. Optimization of Alloy Compositions Using CRC Simulation

**Figure 8:**
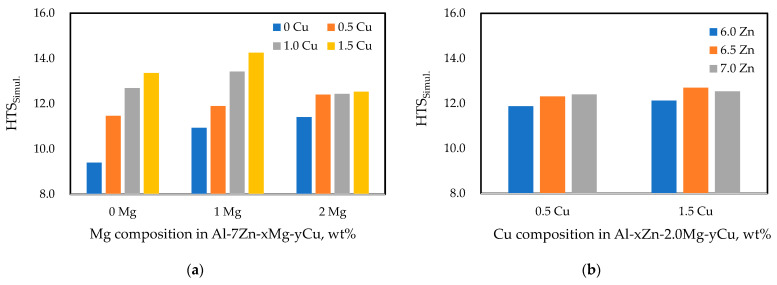
HTS_simul._ values for various Zn, Mg, and Cu contents in Al−Zn−Mg−Cu Alloys: (**a**) Al-7Zn-xMg-yCu; (**b**) Al-xZn-2.0Mg-yCu.

Using the CRC simulation model, HTI simulation was conducted for Al−Zn−Mg−Cu quaternary alloys. In order to ensure a certain level of mechanical properties, the alloy composition was varied within the ranges of Zn 6~7 wt.%, Mg 0~2 wt.%, and Cu 0~1.5 wt.%. The simulated HTI values were applied to derive HTS_simul._, and the results are summarized in Figure 8. The simulation results for various alloy combinations showed that within the current range of Zn compositions, an increase in Zn content slightly increased the HTS_simul._ value, though the magnitude was small. For the Cu element, it is observed that the HTS_simul._ value increased sharply with the increase in Cu content in the Al-7Zn and Al-7Zn-1Mg alloy systems. Similarly, the addition of Mg to the Al-Zn alloy showed an increasing trend in HTS_simul._ values. However, an interesting finding was that with sufficient Mg content (2 wt.%), the increase in HTS was relatively small, even with further increases in Cu content. Furthermore, in the case of the Al-7Zn-2Mg base alloy, adding more than 1 wt.% Cu resulted in lower HTS values compared to alloys without Mg or those with 1 wt.% Mg. From these results, it can be concluded that adding a sufficient amount of Mg is advantageous for reducing the HTS value.

Table 6 summarizes the STRs and f_eutectic_ values of representative alloys with relatively low and high HTS_simul._ among various simulated alloys. In binary Al-Zn alloys, the STR is relatively narrow, resulting in a significantly low HTS_simul._. However, the addition of Mg and Cu to Al-Zn alloys leads to a marked increase in STR and a corresponding increase in HTS. For the ternary Al-Zn-Cu alloys, the STR is wide, but the amount of eutectic liquid, which could fill inter-grain voids caused by solidification shrinkage and thermal contraction, is minimal, thereby significantly increasing HTS. In quaternary alloys with both Mg and Cu added, the STR further increases, and an adequate amount of eutectic liquid forms, which helps to lower the HTS. As the Mg content increases, the STR can increase, but the rise in eutectic liquid can reduce HTS. This trend is consistent with previous studies showing that while HTS increases with STR, the formation of sufficient eutectic phases can mitigate HTS during the final stages of solidification [26,29]. These findings suggest that adding sufficient Mg is highly effective in designing Al−Zn−Mg−Cu alloys with relatively low HTS. Additionally, for the future design of high-strength aluminum casting alloys based on the Al-Zn system, simplifying the alloy composition, as opposed to the conventional quaternary-based alloys developed for wrought applications, may provide valuable insights into developing Al-Zn alloys with sufficiently low HTS.

### 3.4. LPDC for Selected Al−Zn−Mg−Cu Alloys

**Figure 9:**
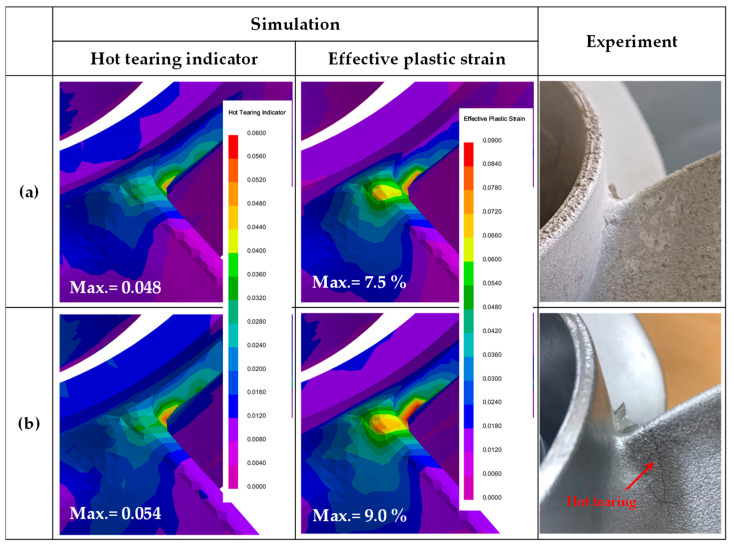
Simulation and experimental results of LPDC Al−Zn−Mg−Cu alloys: (**a**) Al-6Zn-2Mg-0.5Cu; (**b**) Al-6Zn-2Mg-1.5Cu (in wt.%).

Among the simulated alloys, two types of quaternary Al−Zn−Mg−Cu alloys with relatively low HTS values were selected: Al-6Zn-2Mg-0.5Cu alloy, which has the lowest HTS, and Al-6Zn-2Mg-1.5Cu alloy, which can achieve strength improvement due to its higher Cu content despite having a relatively low HTS value. Figure 9 illustrates the simulation results and the actual appearance of prototype propellers manufactured using an LPDC process. The simulation accurately predicted the locations of hot tearing in the actual propellers, showing that higher Cu content correlated with higher HTI and effective plastic strain values. In the actual production process, hot tearing was not observed in the Al-6Zn-2Mg-0.5Cu alloy propellers, whereas in the Al-6Zn-2Mg-1.5Cu alloy propellers, hot tearing frequently occurred at the junction between the hub and the blades, as indicated in Figure 9b. As the amount of Mg and Cu in the alloy increases, it is possible to achieve higher mechanical properties in the product. However, if alloys with high Mg and Cu content are to be manufactured, additional process optimization is required. Therefore, in Part Ⅱ of this study, the effects of various process variables on the occurrence of hot tearing during the LPDC process will be investigated using simulation models, and process optimization will be discussed in detail.

## 4. Conclusions

The development of high-strength aluminum alloys for propeller manufacturing in the leisure boat industry requires a careful balance between mechanical properties and castability. Traditional Al-Si alloys, while cost-effective and easily cast, lack the mechanical robustness required for high-performance applications. Conversely, 7xxx series alloys offer superior mechanical properties but are prone to hot tearing during casting, necessitating optimized alloy compositions. This study demonstrated that adding sufficient Mg to Al−Zn−Mg−Cu alloys can significantly reduce hot-tearing susceptibility, making these alloys suitable for LPDC processes. Through constrained rod casting and finite element simulations, two optimized alloy compositions, Al-6Zn-2Mg-0.5Cu and Al-6Zn-2Mg-1.5Cu, were identified. Experimental validation confirmed the feasibility of these alloys for propeller production, with Al-6Zn-2Mg-0.5Cu showing no hot tearing in prototype castings. However, higher-Cu-content alloys require further process optimization. The findings underscore the potential of 7xxx series alloys in revolutionizing propeller manufacturing, with future research directed toward optimizing casting processes to fully leverage their mechanical advantages.

## Figures and Tables

**Table 1:** Chemical compositions of the alloys fabricated via constrained rod casting (wt.%).

Alloy		Zn	Mg	Cu	Zr	Cr	Si	Fe	Ti	Al
AA7075	Nominal	5.5	2.5	1.5	-	0.2	-	-	-	Bal.
	ICP	5.43	2.37	1.42	-	0.21	0.06	0.17	0.006	Bal.
AA7068	Nominal	8.0	2.5	2.0	0.1	-	-	-	-	Bal.
	ICP	7.66	2.63	1.96	0.11	-	0.03	0.11	0.004	Bal.
AA7055	Nominal	8.0	2.0	2.5	0.1	-	-	-	-	Bal.
	ICP	7.95	1.91	2.50	0.10	-	0.03	0.10	0.005	Bal.

**Table 2:** Factors for the hot-tearing index.

Crack Severity	*f_crack_*	Length (rod)	*f_length_*	Location	*f_location_*
Short hairline	1	51 mm	4	At sprue	1
Full hairline	2	89 mm	8	At ball	2
Crack	3	127 mm	16	Middle of rod	3
Half broken	4	165 mm	32		

**Table 3:** The volume fractions of the eutectic phase, *f_eutectic_*, obtained through experiments and calculations (in %).

Alloy	Experiment	Back Diffusion				Scheil
		10 K/s	20 K/s	30 K/s	50 K/s	
AA7075	2.9, (Ref. [29])	2.62	2.96	3.06	3.25	5.30
Al-7.5Zn-2.4Mg-1.6Cu	3.4, (Ref. [29])	3.74	4.06	4.21	4.39	6.24
Al-6Zn-2Mg-2Cu	3.9 (Ref. [28])	3.27	3.54	3.68	3.86	5.63
Al-9Zn-2Mg-2Cu	4.2 (Ref. [28])	4.62	4.88	5.02	5.20	6.78

**Table 4:** The initial and boundary conditions for LPDC.

Condition	Value
Mold/stalk materials	H13/Al_2_O_3_
Melt supply temperature	720 °C
Initial mold/stalk temperature	350 °C/350 °C
Ambient temperature	20 °C
Inlet pressure	Pressurization: 0→0.3 bar for 8 s Holding: 0.3 bar for 30 s Melt drain: 38 s
Mold/casting heat transfer coefficient	2000 W/m^2^K (Ref. [21])
Mold/air heat transfer coefficient	20 W/m^2^K (Ref. [21])

**Table 5:** Factors influencing the hot-tearing susceptibility.

Alloy	$∆T_{total}$ (°C)	$∆T_{0.9-0.98}$ (°C)	$\frac{∆T_{0.9-0.98}}{∆T_{0.4-0.9}}$	$∆T_{eut.}$ (°C)	$f_{eut.}$ (%)	HTS_exp._
AA7075	156	52.1	0.60	10.5	2.9	328
AA7068	162	17.7	0.14	23.2	5.1	376
AA7055	172	21.8	0.17	27.7	5.3	384

**Table 6:** STR, *f_eutectic_*, and HTS_simul._ values of various simulated alloys.

Alloy	$∆T_{total}$ (°C)	$∆T_{eut.}$ (°C)	$f_{eut.}$ (%)	HTS_simul._
Al-7Zn	43.0	0.0	0.0	9.39
Al-6Zn-2Mg-1.5Cu	166.0	16.7	2.7	12.12
Al-7Zn-1.5Cu	127.0	0.0	0.0	13.36
Al-7Zn-1Mg-1.5Cu	177.0	9.6	1.9	14.25

## Data Availability

The raw data supporting the conclusions of this article will be made available by the authors on request.
